# Severe Dermatologic Toxicity During Amivantamab-Lazertinib Treatment in Epidermal Growth Factor Receptor (EGFR)-Mutated Non-Small Cell Lung Cancer

**DOI:** 10.7759/cureus.115609

**Published:** 2026-09-01

**Authors:** Chloe M Jones, Matthew M Delancy, Steven Bonawitz

**Affiliations:** 1 Otolaryngology, Cooper Medical School of Rowan University, Camden, USA; 2 Plastic and Reconstructive Surgery, Cooper University Health Care, Camden, USA

**Keywords:** amivantamab, cutaneous adverse drug reaction, epidermal growth factor receptor (egfr), lazertinib, non-small cell lung cancer (nsclc)

## Abstract

Amivantamab is an epidermal growth factor receptor (EGFR) and mesenchymal-epithelial transition (MET) antibody. Lazertinib is an EGFR tyrosine kinase inhibitor with central nervous system penetration. Used in combination, this targeted therapy is effective in treating non-small cell lung cancer (NSCLC). However, it has been associated with severe dermatologic adverse reactions.

A 61-year-old woman with a history of EGFR-mutated NSCLC with brain metastases on amivantamab-lazertinib therapy presented for follow-up with drainage from her scalp incision after undergoing a right parietal craniotomy and mass excision five months prior. The patient denied constitutional and neurologic symptoms, but reported yellow staining on her pillow. Examination initially demonstrated yellow fibrinous exudate and hair matting along the surgical incision. Computed tomography (CT) of the head revealed overlying scalp swelling and minimal extra-axial dural thickening over the prior right parietal craniotomy site with stable intracranial metastatic lesions. Due to concern for dehiscence, her biologic therapy was paused, and she was taken to the operating suite where no evidence of infection, delayed healing, or wound dehiscence was noted. A 10 × 5 cm area of patchy granulation tissue and ulceration was observed along the right parietal scalp. Biopsy revealed granulation tissue and multinucleated giant cells and was negative for tumor. Given the risk of malignant progression with all targeted therapy paused, the patient was restarted on amivantamab monotherapy. Following disease progression two months later, she was transitioned to carboplatin and etoposide.

EGFR is widely expressed in skin keratinocytes and intimately involved in epithelial differentiation. Therapies targeting EGFR, such as amivantamab plus lazertinib, are associated with adverse dermatologic reactions, ranging from dry skin to severe scalp ulceration, which can interrupt treatment, contribute to poorer clinical outcomes, and reduce quality of life. Standard of care includes limiting sun exposure and avoiding alcohol-based topical agents. The use of daily prophylactic antibiotics, such as oral doxycycline, topical clindamycin, chlorhexidine wash, and ceramide moisturizer, may significantly reduce the risk of adverse skin reactions. Implementation of this multicomponent regimen reduced the rate of severe scalp reactions from 26% to 10% relative to the standard of care, with sustained benefit observed for at least six months.

## Introduction

Lung cancer is the leading cause of cancer-related deaths, with non-small cell lung cancer (NSCLC) representing 85% of all cases [1]. Globally, an estimated 23.9% of NSCLCs are associated with activating epidermal growth factor receptor (EGFR) mutations [1]. Incidence varies substantially with ethnicity, ranging from 38.8% in Asian populations to 17.4% in Caucasian populations [1]. EGFR mutations have also demonstrated higher prevalence in female nonsmokers [1]. As a result, EGFR inhibitors have become increasingly utilized for the treatment of NSCLC, as these drugs have been shown to improve progression-free survival compared to standard chemotherapy [1].

Amivantamab is an EGFR and mesenchymal-epithelial transition (MET) antibody, while lazertinib is an EGFR tyrosine kinase inhibitor with central nervous system (CNS) penetration [2-4]. Osimertinib monotherapy and osimertinib in combination with a platinum agent are first-line therapies for NSCLC associated with exon 19 deletion or L858R mutations [5]. Newer agents such as amivantamab plus lazertinib have been shown to improve median progression-free survival in this population; however, EGFR-targeted therapies are associated with a higher rate of discontinuation due to drug-related adverse effects [5-6]. Interruption of therapy due to adverse effects was reported as high as 10% for amivantamab plus lazertinib compared to 3% for osimertinib [3,5,6]. EGFR inhibitors have been associated with drug-related adverse effects, including cutaneous reactions, diarrhea, and transaminitis [2]. EGFR is involved in the growth, maturation, and differentiation of epidermal and epithelial cells [4]. While tumors with EGFR mutations respond positively to targeted therapies, the wide expression of EGFR in skin keratinocytes contributes to cutaneous reactions as keratinocyte homeostasis is disrupted [4,7]. Rash, paronychia, and pruritus are common dermatologic adverse reactions, while scalp findings are rare [3]. Furthermore, previous rare reports of scalp involvement describe a preceding papulopustular rash on the face and trunk, followed by diffuse scalp involvement [7]. In the present case, we discuss a patient with a rare, severe dermatologic scalp reaction to amivantamab plus lazertinib, characterized by delayed onset and focal involvement of a prior surgical site. Treatment of this reaction required multidisciplinary management and resulted in oncologic treatment interruption.

## Case presentation

We present the case of a 61-year-old woman with a past medical history of NSCLC associated with an EGFR gene exon 19 mutation that was diagnosed at age 53. At the time of diagnosis, she was initially treated with a left lower lobectomy; however, one year later, she developed local recurrence and underwent wide local excision. Three years later, imaging demonstrated metastasis to the regional lymph nodes, and she was treated with first-line osimertinib. This was followed by gamma knife stereotactic radiosurgery after the development of brain metastasis. After further disease progression, she completed a course of second-line carboplatin, pemetrexed, and pembrolizumab. Given further progression the following year, she was transitioned to amivantamab and lazertinib. During this treatment course, she underwent right craniotomy due to an increase in size of the brain metastases and associated radiation necrosis, and she received radiation therapy to the left chest for lung nodule progression. Five months following craniotomy, she presented with increased drainage from the scalp. At the time of presentation, the patient denied fever, chills, headache, or focal neurologic deficits but endorsed yellow staining on her pillow corresponding to the incision site.

The patient was found to be afebrile with no evidence of leukocytosis. Physical examination revealed yellow fibrinous exudate and eschar along the incision without surrounding erythema. Given limited visibility due to hair matting and concern for wound dehiscence or infection, EGFR-targeted therapies were paused. Computed tomography (CT) of the head revealed overlying scalp swelling and minimal extra-axial dural thickening over the prior right parietal craniotomy site with stable intracranial metastatic lesions. The patient was prescribed doxycycline and taken to the operating room by neurosurgery and plastic surgery for wound debridement. After hair removal, irrigation, and debridement of the incision site, there was no evidence of wound dehiscence. The prior incisions were intact and well approximated with appropriate healing. There was no pus or surrounding erythema to indicate active infection intraoperatively. A 10 × 5 cm area of patchy granulation tissue and ulceration with mild bleeding was visible around the prior surgical site (Figure 1). 

**Figure 1:**
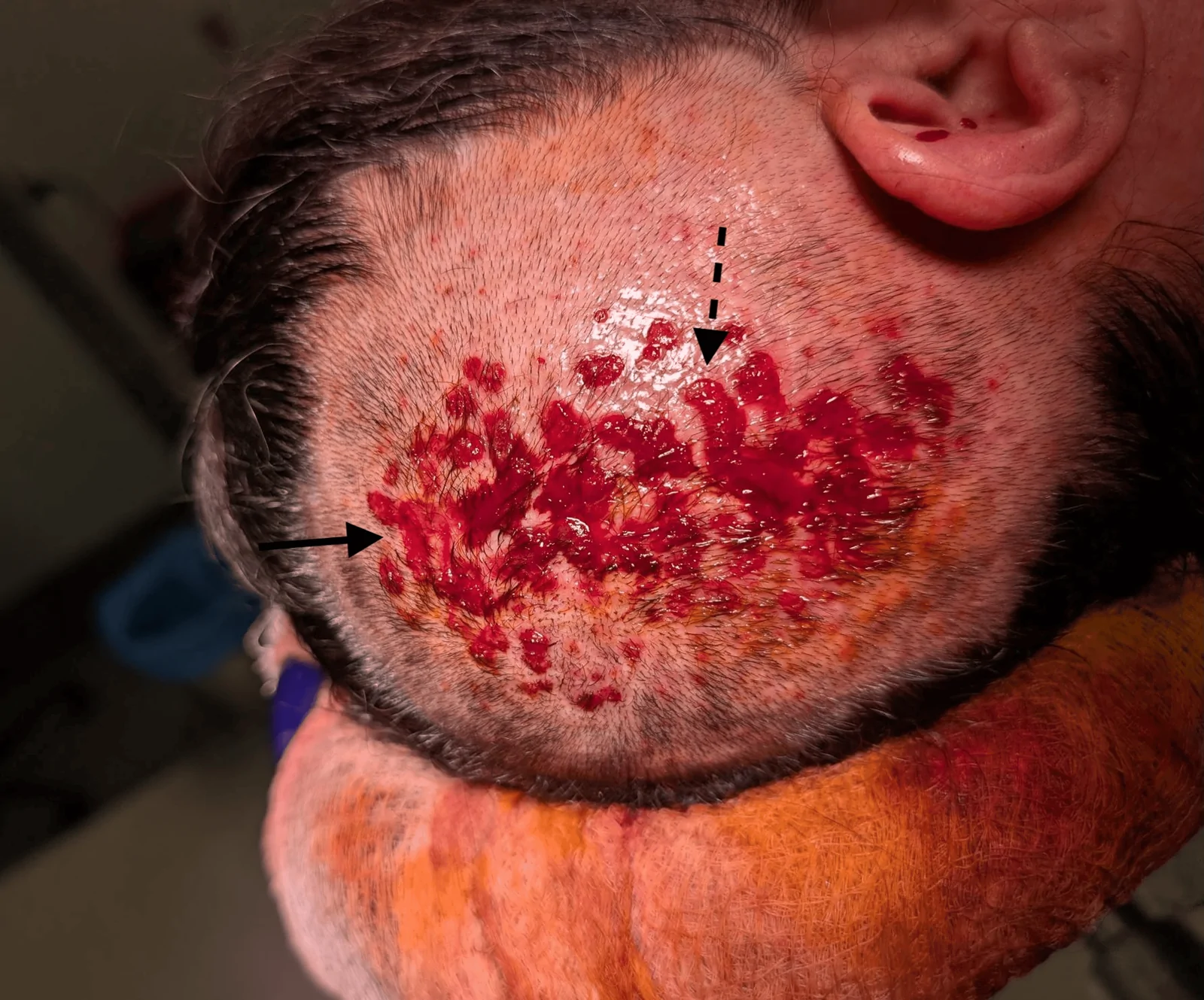
Intraoperative photograph demonstrating an approximately 10 × 5 cm area of abnormal scalp tissue with a honeycomb appearance Solid arrows indicate representative areas of ulceration with mild bleeding, and dashed arrows indicate areas of granulation tissue.

An incisional scalp biopsy was taken, the site was closed, and a sterile dressing was placed. Pathology revealed granulation tissue and multinucleated giant cells (Figure 2). Testing of the site was negative for herpes simplex virus (HSV) 1, HSV 2, and varicella zoster virus. While the patient had previously undergone gamma knife radiation for brain metastases, this treatment had been completed two years prior without adverse effects and was considered unlikely to be contributory to the current cutaneous lesion. Lazertinib therapy continued to be withheld due to concerns of impaired wound healing. Rather than withholding her entire treatment regimen, the patient was continued on amivantamab monotherapy based on clinical judgment. The patient was discharged with a course of oral acyclovir and mupirocin ointment for application to the incision site.

**Figure 2:**
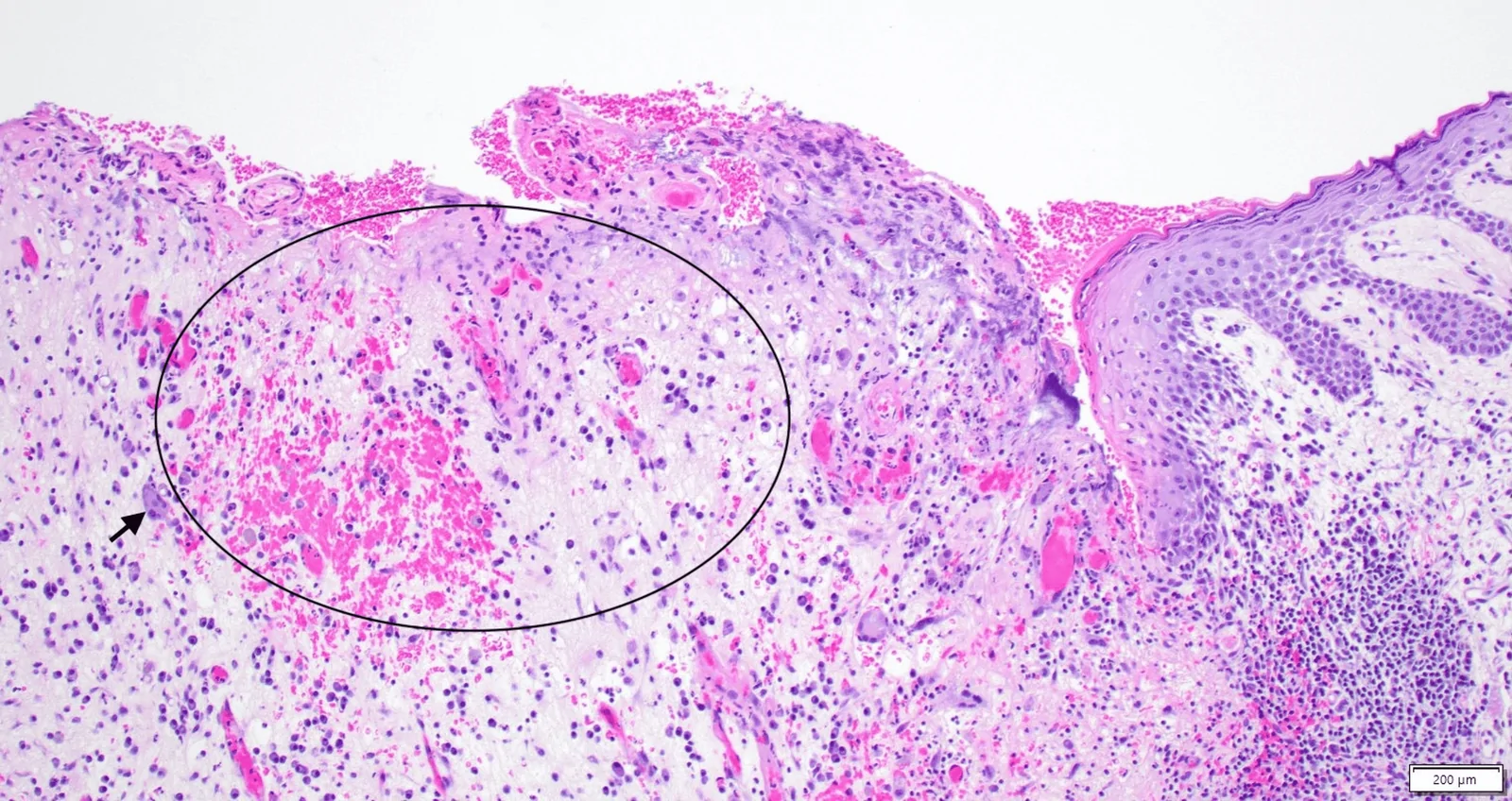
Histopathologic examination of incisional scalp biopsy demonstrating granulation tissue and multinucleated giant cells Arrows identify representative multinucleated giant cells, and circles indicate surrounding granulation tissue (hematoxylin and eosin stain; original magnification ×40; scale bar = 200 μm).

One month later, on exam, the scalp incision site appeared healed with no residual drainage or erythema. During a follow-up visit one month later, the patient was found to have further disease progression with transformation to small cell carcinoma. The patient was transitioned to carboplatin and etoposide therapy and continues to follow with oncology.

## Discussion

EGFR is widely expressed in skin keratinocytes and is involved in epidermal and epithelial differentiation [4]. EGFR-targeted therapies, such as amivantamab plus lazertinib, are associated with adverse dermatologic reactions, including rash, paronychia, dry skin, and severe ulceration [2]. Scalp ulceration is a rare complication of EGFR-targeted therapies. One prior case series by Zhou et al. described nine patients who developed severe scalp ulceration, where four patients were treated with amivantamab and five received amivantamab plus lazertinib [7] (Table 1). Evidence of scalp findings in patients with amivantamab alone may suggest that amivantamab may be associated with these dermatologic reactions [7]. Within three weeks of drug initiation, all patients in their series first developed a papulopustular rash on the face and trunk, followed by ulcerative plaques on the scalp by 23 weeks, leading to drug discontinuation in six patients [7]. Further, on follow-up, Zhou et al. reported that ulcerative lesions took over three months to heal and were associated with scarring and alopecia, negatively contributing to long-term quality of life for patients [7]. Out of all antibiotic and steroid therapies attempted, patients reported symptomatic relief with prednisone therapy only; however, ulceration worsened with dose tapering [7].

**Table 1:** Clinical course and outcomes of the present case compared with amivantamab-associated scalp ulcerations in a prior case series (n = 9)

	Present case (n = 1)	Zhou et al. [7] (n = 9)
Therapeutic agent	Amivantamab-lazertinib	Amivantamab: n = 4; amivantamab plus lazertinib: n = 5
Preceding rash	Absent	Present; described as a papulopustular rash on the face and trunk (chest, back, suprapubic region) within three weeks of drug initiation
Time from treatment initiation to scalp reaction	7 months	<23 weeks
Scalp lesion distribution	Focal; right parietal scalp overlying prior surgical site	Diffuse
Scalp lesion description	Area of patchy granulation tissue and ulceration with mild bleeding	Follicular pustules with progression to pustular and ulcerative plaques
Oncologic treatment interruption	Yes; lazertinib withheld, followed by amivantamab discontinuation due to disease progression	Yes; amivantamab discontinuation due to scalp ulceration (n = 6) or disease progression (n = 3)
Treatment of scalp lesions	Doxycycline, acyclovir, and topical mupirocin	Topical and systemic antibiotics and steroids; no relief with antibiotics; benefit with prednisone
Healing time	1 month	>3 months
Permanent complications	None	Follicular erythema; scarring alopecia

In contrast, our patient first developed scalp ulceration over seven months following initiation of amivantamab-lazertinib therapy. She reported no preceding bodily rash or additional cutaneous symptoms, instead presenting only with drainage overlying her prior surgical site. Association with a prior incision may lead to delayed recognition of a drug-related reaction, as initial concerns were of wound dehiscence or infection. In addition, presence of patient hair contributes to limited view of scalp lesions, which may further delay recognition of dermatologic drug reactions. One month following biopsy, our patient demonstrated a well-healed incision site on exam with no evidence of further drainage or ulceration. She completed a course of oral acyclovir and mupirocin ointment, achieving adequate healing with no complications such as continued drainage, cutaneous progression, or bacterial superinfection.

Severe dermatologic reactions can interrupt treatment, limit their use, and contribute to poorer clinical outcomes and reduced quality of life [3]. Patients on EGFR inhibitors should be monitored for dermatologic changes, as early recognition of adverse reactions can improve outcomes and prevent complications. Open wounds with prolonged healing should be monitored for signs of bacterial superinfection. Standard of care involves general guidance such as limiting sun exposure and alcohol-based topical agents [2,4]. However, use of multicomponent prophylactic regimens composed of antibiotics such as 100 mg oral doxycycline or topical 1% clindamycin, 4% chlorhexidine wash, and ceramide moisturizer have been shown to significantly reduce the risk of adverse skin reactions [2]. One prior study has reported that implementation of this combination regimen reduced the rate of scalp reactions from 26% to 10% relative to the standard of care, with sustained benefit observed for at least 6 months [2]. However, further study is required to contextualize the use of prophylactic measures for scalp involvement specifically. While scalp involvement is a rare complication of amivantamab-lazertinib therapy, it is also varied in its presentation and onset, thus oncologic providers should be aware of potential complications. 

## Conclusions

While NSCLC is associated with poor prognosis, EGFR inhibitors are effective treatments and have been shown to prolong progression-free survival. However, severe adverse reactions such as dermatologic ulceration may lead to treatment interruptions and limit therapeutic benefit, resulting in poorer clinical outcomes. This case highlights a rare presentation of amivantamab-lazertinib toxicity requiring physician awareness of the adverse effects of EGFR-targeted therapies. Further research is indicated to identify optimal prevention and management strategies for patients with cutaneous reactions to EGFR-targeted therapies.
